# A Validated HPLC-UV-ESI-IT-MS Method for the Quantification of Carnosol in *Lepechinia mutica*, a Medicinal Plant Endemic to Ecuador

**DOI:** 10.3390/molecules28186701

**Published:** 2023-09-19

**Authors:** Natalí Solano-Cueva, Jorge G. Figueroa, Corina Loja, Chabaco Armijos, Giovanni Vidari, Jorge Ramírez

**Affiliations:** 1 Departamento de Química, Universidad Técnica Particular de Loja, Loja 1101608, Ecuador; nesolano@utpl.edu.ec (N.S.-C.); jgfigueroa@utpl.edu.ec (J.G.F.); cbloja@utpl.edu.ec (C.L.); cparmijos@utpl.edu.ec (C.A.); 2 Medical Analysis Department, Faculty of Science, Tishk International University, Erbil 44001, Iraq; vidari@unipv.it

**Keywords:** carnosol, *Lepechinia mutica*, HPLC-UV-ESI-IT-MS analysis, analytical method validation

## Abstract

The diphenolic diterpene carnosol was isolated from several species of the family Lamiaceae, including *Lepechinia mutica*, a medicinal plant endemic to Ecuador. The compound has exhibited high antioxidant, anti-inflammatory, antimicrobial, neuroprotective, and antifungal properties, as well as promising cytotoxicity against prostate, breast, skin, leukemia, and human colon cancer cell lines. In this paper, we developed and validated a simple, accurate, and reliable analytical HPLC-UV-ESI-IT-MS method, carried out on a C18 column, which is potentially suitable to quantify carnosol in plant extracts. The procedure complied with the established ICH validation parameters of analytical range (linearity in the range of 0.19–5.64 μg/g dried leaves; RE_AVERGE_ = 4.9%; R^2^ = 0.99907), analysis repeatability (RSD = 2.8–3.6%), intermediate precision (RSD = 1.9–3.6%), accuracy (estimated as % carnosol recovery in the range of 81 to 108%), and robustness. Finally, the LOD (0.04 µg/mg) and LOQ (0.19 μg/mg) values of carnosol/dried leaves were determined. Using this validated method, the content of carnosol in *L. mutica* was estimated to be 0.81 ± 0.04 mg/g of dried leaves (0.081%).

## 1. Introduction

**Figure 1:**
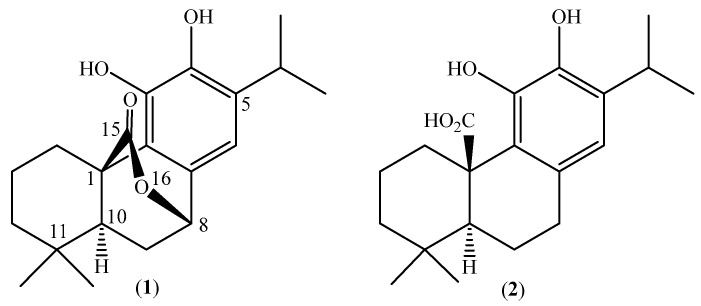
Chemical structures of carnosol (**1**) and carnosic acid (**2**).

Over 78 years ago, an intensely bitter principle was first isolated from sage, *Salvia carnosa* Dougl., and named carnosol (syn. picrosalvin). Later, Wenkert and collaborators firmly established structure **1** (Figure 1) for this *o*-diphenolic hydrophenanthrene diterpene lactone of the abietane ferruginol type, with the molecular formula C_20_H_26_O_4_ [1] and IUPAC name (1*R*,8*S*,10*S*)-3,4-dihydroxy-11,11-dimethyl-5-propan-2-yl-16-oxatetracyclo [6.6.2.0^1,10^.0^2,7^]hexadeca-2,4,6-trien-15-one. Carnosol (**1**) is probably an oxidative derivative of carnosic acid (**2**) [2], and it occurs abundantly in the extracts of leaves and aerial parts of several Lamiaceae, such as *Rosmarinus officinalis* (rosemary) [2], *Salvia officinalis* [2], *S. eremophila* Boiss [3] and other *Salvias* [4], *Sphacele chamaedryoides* [5], *Lepechinia hastata* [6], and *Lepechinia mutica* [7]. Carnosol has shown a wide variety of interesting biological activities. In fact, it has shown significant antibacterial effects against Gram-positive bacteria, including two penicillin- and ampicillin-resistant *Staphylococcus aureus* strains [6], strong inhibitory effect on EBV-EA induction, and remarkable activity on mouse skin tumor promotion in an in vivo two-stage carcinogenesis model. The antiproliferative activity of carnosol has been determined against A-549 (human lung carcinoma), HeLa (human carcinoma of the cervix), Hep-2 (human carcinoma of the larynx), and MCF-7 (human breast adenocarcinoma) cell lines, and against a Vero (African green monkey kidney) cell line [8]. Carnosol exhibits higher cytotoxicity against gastric tumor AGS cells than normal fibroblasts [5]. Moreover, carnosol induces apoptosis and prevents the growth of new vessels from tumors. This diterpene also contributes to the high antioxidant and anti-tumorogenesis properties of rosemary (*Rosmarinus officinalis* L.), and it is efficacious in the chemoprevention and/or chemotherapy of hormone-responsive cancers [4,8,9,10]. Carnosol (**1**) also inhibited neuroinflammation and produced anti-inflammatory effects in models of rheumatoid arthritis. The cytotoxicity and anti-inflammatory properties seem to be associated with the modulation of multiple deregulated pathways, including NF-kB, phosphatidylinositol-3-kinase, androgen and estrogen receptors, and PKC [10], and inhibitory effects on prostaglandin PGE_2_ formation [11]. Finally, carnosol showed potent antifungal activity against the dermatophyte *Microsporum canis* and against *Pyricularia oryzae* [7]. This fungus is responsible for blast disease, also known as rice rotten neck, which is the most important disease in rice cultures worldwide.

Although clinical uses of *L. mutica*, as well as carnosol (**1**), have not been reported so far, compounds **1** and **2** are used as antioxidant preservatives in food and nonfood products, where they are labeled “extracts of rosemary” (E392) [2]. Moreover, a prototype pharmacokinetic study was recently carried out with the determination of carnosol (**1**) in the plasma samples of a healthy volunteer after administration of rosemary extract [12].

Our research group isolated carnosol (**1**) during an extensive investigation of volatile and non-volatile constituents of extracts from *Lepechinia mutica* (Benth.) Epling and other *Lepichinia* species [7,13,14,15]. *L. mutica* is endemic to Ecuador [16,17], where the leaves are used as a traditional remedy to treat headache and nervous affections [18,19]. In addition to the significant antifungal properties [7], we found that carnosol exhibited selective inhibitory activity against butyrylcholinesterase with an IC_50_ value less than that of the well-known ChE inhibitors donepezil and allicin. These results make carnosol a potential therapeutic for the treatment and/or the prevention of AD [13,20].

The unique profile of biological activities displayed by carnosol (**1**) and the widespread ethnomedical uses [2] make the implementation and validation of a simple and reliable analytical method to quantify this diterpenoid in plant extracts highly desirable. In this paper, we describe an accurate high-performance liquid chromatography (HPLC)–UV-diode array detection (DAD)–electrospray ionization (ESI)–ion trap (IT)–mass spectrometry (MS) procedure, which we believe to be of general applicability for the quantification of carnosol in plant extracts. As a proband example, we quantified the content of carnosol (**1**) in an EtOAc extract of *L. mutica*.

HPLC-UV-ESI-IT-MS is an analytical technique with great versatility, sensitivity, and analytical power, where the individual capabilities of each technique are enhanced synergistically. While liquid chromatography separates mixtures of many components, mass spectrometry provides spectral information that may help to identify or confirm the suspected identity of each separated component. This hyphenated technique is thus widely used to separate, identify, and quantitate different secondary metabolites occurring in foods, drugs, plant extracts, and other biological matrices [21]. HPLC-MS is especially suitable for the analysis of most polar and thermally labile metabolites that cannot be processed by gas chromatography–mass spectrometry. 

## 2. Results and Discussion

### 2.1. Specificity of the Method

A sample of carnosol (**1**) was isolated from an EtOAc extract of *Lepechinia mutica* leaves following the procedure described in a previous paper [7]. After crystallization, purified carnosol was used as the standard in the subsequent experiments. The identity of the isolated sample was confirmed by IR, NMR, UV spectra, and a comparison with the literature [1,7,22]. 

**Figure 2:**
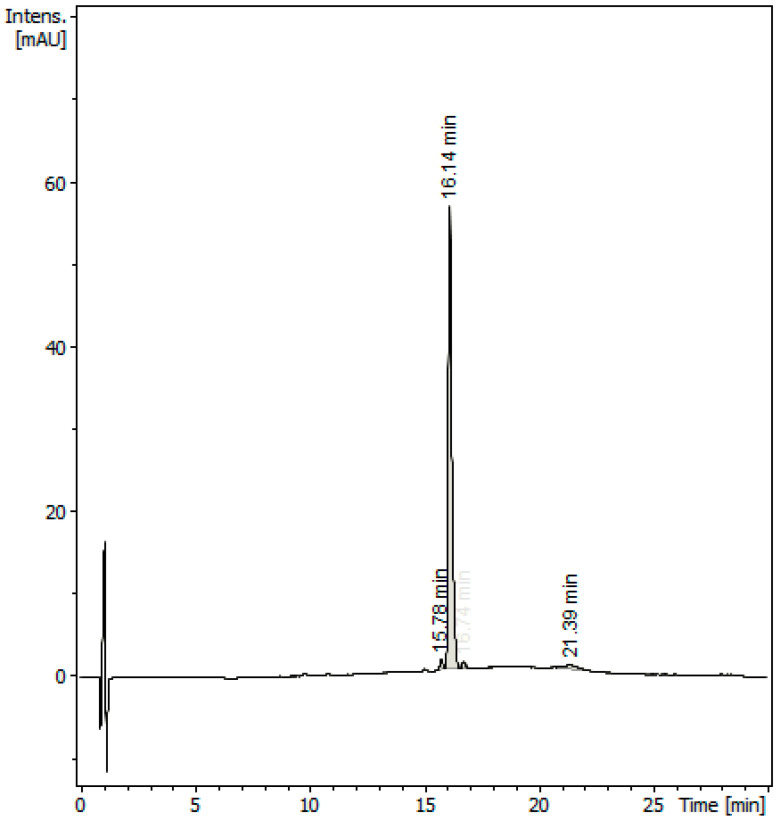
HPLC chromatogram of purified carnosol (**1**) isolated from *Lepichinia mutica*.

To get the best separation efficiency, different HPLC columns, compositions and elution time of the mobile phase, and column temperatures were tested and compared. Under optimized HPLC conditions (see Section 3.4), the chromatogram of the purified sample (Figure 2) showed a major peak, eluted with a retention time of 16.14 min, due to carnosol, which was flanked by two minor peaks, with a retention time of 15.78 min and 16.74 min, respectively. The first minor peak was tentatively identified as rosmarinic acid from the [M + H]^+^ ion at *m*/*z* 361, while the other peak was unidentified. The peaks in the chromatogram were highly symmetric and base-separated. Moreover, the duration of the analysis was reasonable. 

**Figure 3:**
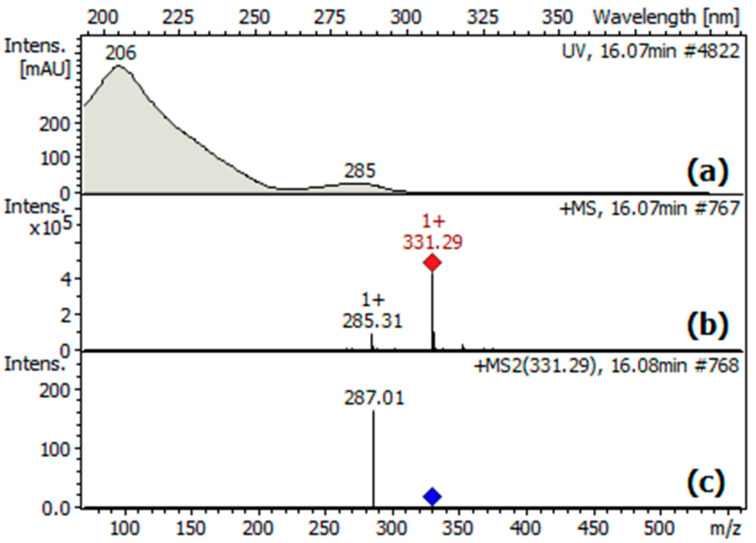
(**a**) UV absorption spectrum; (**b**) ESI-MS spectrum; (**c**) ESI-MS^2^ spectrum of carnosol (**1**).

The identity of the major peak in the chromatogram as carnosol (**1**) was confirmed by the UV absorption spectrum (Figure 3a), which showed two characteristic bands with maximum absorbances at about 210 and 285 nm. Moreover, the UV spectra taken at the peak upslope and downslope, normalized, and compared, were identical, indicating that the peak was spectrally pure or that the peak corresponded to a single compound. On the other hand, the ESI-MS spectra, taken at different points of the peak, consistently showed a pseudo-molecular ion peak [M + H]^+^ at *m*/*z* 331.29 [C_20_H_27_O_4_]^+^ (Figure 3b), in accordance with the MW = 330.42 g/mol of carnosol (C_20_H_26_O_4_). Moreover, the ESI-MS^2^ spectrum (Figure 3c) showed an ion at *m*/*z* 287.01, corresponding to the loss of CO_2_ from the [M + H]^+^ ion. 

Analyses conducted at regular time intervals indicated that the sample of the standard carnosol was stable and did not degrade under normal experimental conditions for the entire time of the experiments. Moreover, the purity of the standard was estimated to be 94.22% from the HPLC chromatogram (Figure 2).

### 2.2. Linearity Study of Standard Carnosol

The linearity of an analytical procedure is the ability to obtain a response that is directly proportional to the concentration (amount) of the analyte in samples within a given range [23,24,25,26]. To determine the linearity of our method, a series of methanol solutions of the standard carnosol (**1**) were prepared, covering the range of 1–300 μg/mL. The corresponding areas of the UV absorption band at about 285 nm were electronically calculated, and the resulting graph (mAU vs. concentration) yielded the coefficient of determination, R^2^ = 0.99907 (see Figure S1 in the SI). However, R^2^ alone is not adequate to demonstrate linearity, since values greater than 0.999 can result even from data exhibiting curvature. Therefore, additional criteria are needed to confirm linearity [27,28], and % *RE* values of 15–20% were suggested as an acceptance criterion for linearity [28]. Since carnosol concentrations within the range of 2–5 μg/mL were associated with % *RE* values ranging from 84 to 226, carnosol analysis was considered linear in the range of 10–300 μg/mL, where the values of % *RE*_AVERAGE_ and % *RE*_MAX_ were determined as 4.9 and 17.1, respectively. 

### 2.3. Lowest Limit of Detection (LOD) and Lowest Limit of Quantitation (LOQ) of Standard Carnosol

The lowest limit of detection (LOD) [23,24,25,26] is the lowest quantity of a substance that can be distinguished from the absence of that substance (a blank value) with a certain confidence level (generally 99%). In other words, the LOD value is the concentration of an analyte that gives rise to a signal statistically significantly larger than the signal arising from the repeated measurements of a blank. Thus, it is the lowest concentration of an analyte in a sample that can be detected but not necessarily quantified. On the other hand, the LOQ value is defined as the lowest concentration of an analyte that can be quantitatively determined with acceptable precision and accuracy. The limit of carnosol detection (LOD) was determined to be 2 μg/mL, based on a signal-to-noise ratio of 3.4. Furthermore, the limit of carnosol quantification (LOQ) was set at 10 μg/mL, which corresponded to the lowest concentration at which linearity was observed. These results demonstrate that the HPLC-UV-ESIMS method is sensitive for the quantitative determination of carnosol in plant extracts.

### 2.4. Quantification of Carnosol in Lepechinia mutica

**Figure 4:**
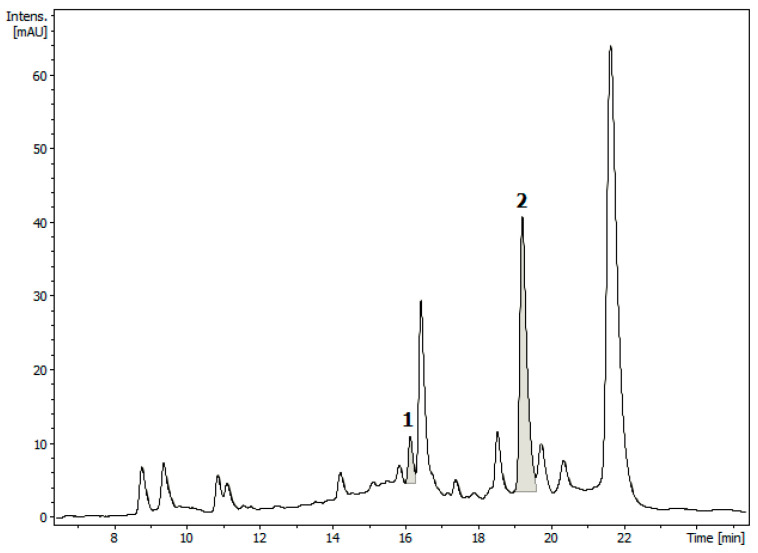
HPLC chromatogram of a chlorophyll-free residue (A’) from an EtOAc extract of dried leaves of *Lepechinia mutica*. Peak 1: carnosol (**1**); peak 2: carnosic acid (**2**).

Having demonstrated good linearity for the reference sample, we estimated the content of carnosol (**1**) in an EtOAc extract of dried leaves of *L. mutica* using the same optimized HPLC conditions as those used for the standard. A sample of the residue resulting from evaporation of the extract was redissolved in MeOH-H_2_O, 9:1 *v*/*v*, freed from chlorophylls, and analyzed in three replicates. Carnosol was eluted with a retention time of 16.17 ± 0.04 min (Figure 4). The UV and ESI-MS spectra taken at the peak upslope and downslope revealed peak homogeneity. The mean concentration of carnosol (**1**), estimated using the calibration curve, was 0.81 ± 0.04 mg/g (0.081% *w*/*w*) of the dried leaves. Thus, the calculated linear range, LOD, and LOQ of carnosol were 0.19–5.64, 0.04, and 0.19 mg/g of the dried leaves, respectively. 

The intense peak 2 in the HPLC chromatogram (Figure 4), with a retention time of 19.25 min, was tentatively assigned to carnosic acid (**2**) from the UV spectrum and the [M + H]^+^ ion at *m*/*z* 333, which was in accordance with the MW = 332.44 g/mol of compound **2** (C_20_H_28_O_4_). However, the lack of a standard in our hands prevented us from developing a validated quantification method for carnosic acid in the extract.

### 2.5. Analytical Method Validation

To validate the analytical method, the following parameters were determined according to the IUPAC and the International Conference on Harmonization (ICH) guidelines [23,24]: precision (repeatability and intermediate precision), accuracy, and robustness.

#### 2.5.1. Precision

The precision of an analytical procedure is defined as the closeness of agreement (degree of scatter) between a series of measurements obtained from multiple samplings of the same homogeneous sample under the prescribed conditions [23,24,25,26]. Precision may be considered at the levels of repeatability and intermediate precision.

##### Repeatability

Repeatability is a measure of the variability in results experienced by a single analyst on a single instrument using the same operating conditions over a short timescale [23,24,25,26]. During validation, repeatability is performed by analyzing multiple replicates of a homogeneous sample using the analytical method. The mean and the standard deviation (SD) are calculated, and the precision is expressed as the relative standard deviation (RSD), given by Equation (1):

(1) $$\mathrm{RSD}\;\left(\%\right)=\frac{\mathrm{SD}\times 100}{\mathrm{Mean}}$$

In our experiments, three samples (5, 10, and 15 mg) of residue A’ from the EtOAc extract of dried leaves were analyzed separately in three consecutive days by an analyst (analyst 1), following the procedure described in Section 2.4. The concentration (mg/g) of carnosol (**1**) was determined in each analysis by interpolation of the calibration curve. The repeatability of the method was determined to be excellent, yielding a calculated relative standard deviation (RSD) ranging from 2.8 to 3.6% (see Table S1 in the SI). This range comfortably falls within the AOAC’s recommended value of approximately 3.7% for analyte concentrations below 1 mg/g, indicating the reliability of the method [29]. Moreover, the analysis of variance (ANOVA) indicated no significant difference between the results, as evidenced by an F-value of 2.87 and a *p*-value of 0.134.

##### Intermediate Precision

In analytical method validation, ‘intermediate (measurement) precision’, sometimes referred to as ‘within-laboratory reproducibility’, gives an estimate of the variation in results when measurements are made in a single laboratory but under conditions that are more variable than repeatability conditions. The aim is to obtain a precision estimate that reflects all sources of variation that may occur in a single laboratory under routine conditions (different analysts, extended timescale, different pieces of equipment, etc.) [23,24,25,26]. To validate our analytical method, intermediate precision was estimated by comparing the results obtained by analyst 1 (see Section Repeatability) with those obtained by another analyst (analyst 2), who quantified carnosol in three samples (5, 10, and 15 mg) of residue A’ in three consecutive days under identical experimental conditions. The relative standard deviations (RSDs) ranged between 1.9 and 3.6% (see Table S2 in the SI). 

The ANOVA results indicated that there were no significant differences between the results of the two analysts across three concentrations spanning the analytical range of the method. Moreover, these RSD values were less than the recommended reproducibility threshold of 6% established by the Association of Official Agricultural Chemists (AOAC) [29]. These findings provided confidence in the intermediate precision of the method, making it suitable for use in future studies and applications.

#### 2.5.2. Accuracy (% Recovery)

The accuracy, sometimes termed trueness, of an analytical procedure expresses the nearness (closeness of agreement) between the value that is accepted as an accepted reference value and the measured value. Accuracy can also be described as the extent to which the test results generated by the method and the true (or accepted) reference value agree [23,24,25,26]. 

**Figure 5:**
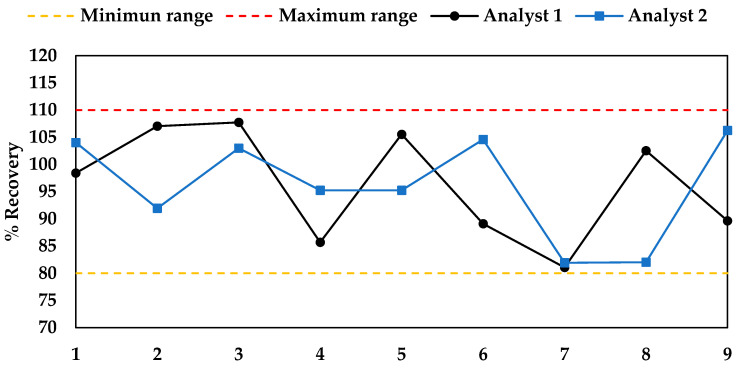
Graphs of the carnosol % recovery (% R) for the two groups of analyses in the accuracy test.

The accuracy of the developed method was evaluated by adding known amounts of standard carnosol to samples of *L. mutica* extract and comparing the extracted amounts to the added amounts. Each of the two analysts 1 and 2 carried out nine experiments in three consecutive days (see Section 3.8.2). The graphs of the percent carnosol recovery (% R) for the two groups of analyses (Figure 5) showed a normal distribution of values, ranging from 81% to 108%, with a mean value of 96% over the entire range. These values were within the range acceptable by the AOAC validation guidelines [29], indicating that the method was deemed precise and accurate. 

#### 2.5.3. Robustness

The robustness of an analytical procedure is a measure of its capacity to remain unaffected by small but deliberate variations in method parameters, and it provides an indication of its reliability during normal usage [24]. Among the large number of potentially variable parameters, we assumed flow rate, temperature, UV detection wavelength, mobile phase composition, and gradient changes to be the most reasonably variable parameters in the analytical conditions. The resolution of 0.8 between the peaks of rosmarinic acid and carnosol was considered the most important performance characteristic of the method. According to the one-factor-at-a-time (OFAT) approach [30], the selected parameters were changed one by one in both directions from the nominal (optimal) value. The effects of the changes in the parameters on the peak resolution were monitored by recording the HPLC chromatogram for each experiment. The results are reported in Table 1. 

The RSD values among measurements ranged from 2.7 to 4.9% (Table 1), which indicated the robustness of the method.

## 3. Materials and Methods

### 3.1. General Information

All solvents were reagent grade or HPLC grade and were purchased from Sigma-Aldrich (St. Louis, MO, USA). Formic acid was supplied by Sigma-Aldrich. A high-performance liquid chromatography (HPLC) Dionex UltiMate 3000 instrument, with a photodiode array detector model DAD-3000 (RS), coupled to an Amazon Speed Ion-Trap mass spectrometer analyzer (AmaZon speed, Bruker, Billerica, MA, USA) with an electrospray ionization source (ESI), was used. The system was controlled using Bruker Daltonics HyStar 3.2 software with an Ethernet data interface. An Agilent Technology Eclipse Plus stainless steel column (Santa Clara, CA, USA) filled with a C18 reversed phase, particle size 5 μm, internal diameter 2.1 mm, and length 150 mm, was used in all the HPLC separations. The UV diode array detector (DAD) was set at 285 nm.

### 3.2. Plant Material

*L. mutica* (Benth.) Epling leaves were collected in *Cerro Villonaco*, in the province of Loja (geographic coordinates: 693096 E–9557752 N), Ecuador, in June 2018, with authorization from the Ministry of Environment of Ecuador (MAE) (authorization no. MAE-DNB-CM-2016-0048). A voucher specimen (accession number PPN-la-005) was deposited at the Herbarium of the Universidad Técnica Particular de Loja (HUTPL). 

### 3.3. Standard Carnosol

Three lots of freshly collected leaves of *L. mutica* (600 + 400 + 200 g) were separately dried at 35 °C under forced air circulation. Subsequently, each dried lot was ground in liquid nitrogen and soaked in EtOAc (1.2 L) for 1 h at room temperature, using an ultrasonic bath at 35 °C in the last 15 min. The extraction was repeated three times for each lot to obtain, after solvent evaporation, three dense oily residues. They were pooled together to provide a total of 24.1 g (residue A). Subsequently, raw carnosol (1, 38.47 mg, 0.015% *w*/*w* dry leaves) was isolated from a sample of A (1 g) by medium-pressure column chromatography, according to the procedure described in a previous paper [7]. Double recrystallization from EtOH yielded purified **1** as a white powder (5.12 mg, 0.0020% *w*/*w* dry leaves), which was used as a working standard in the experiments. The identity of isolated carnosol was confirmed using IR NMR, UV, and MS spectra (Figure 3), and comparison with the literature [1,7,22]. A purity of 94.22% was estimated for the standard by comparing the area of the peak at 16.14 min with the total area of the peaks in the HPLC-MS chromatogram (Figure 2), without applying a correction factor. For preparing each test solution, the carnosol concentration was adjusted according to the purity of the standard. The standard carnosol (**1**) was stored under N_2_ in sealed amber vials at −15 °C until analyses, which were performed within three weeks after the storage. 

### 3.4. HPLC Analysis of Standard Carnosol 

Different mobile phases, column types, and stationary phase size combinations, as well as different flow rates and column temperatures, were tested to obtain the most efficient chromatographic separation of carnosol from impurities. Under optimized HPLC conditions, the mobile phase was constituted of two mixtures: A (30% acetonitrile/70% water/0.1% formic acid) and B (60% acetonitrile/40% water/0.1% formic acid). They were mixed according to the following gradient program: 100% A, from 0 to 4 min, followed by 50% A/50% B, from 4 to 17 min, followed by 100% B, from 17 to 20 min, followed by 100% A, from 20 to 25 min. The flow rate and injection volume in each analysis, carried out at 22 °C, were 0.5 mL/min and 0.5 μL, respectively. With this method, the retention time of carnosol (**1**) was 16.14 ± 0.04 min (Figure 2).

### 3.5. MS Chromatographic Conditions

The ionization was set in positive mode, with the following parameters: voltage of the ionization capillary: 4.500 V; mass range: *m*/*z* 100–2000; nebulizer set at 26.0 psi; nitrogen drying temperature: 200 °C; dry gas (N_2_): 6.0 L/min; rolling averages: 2 cts; averages: 2, ICC (ion charge control): on. The instrument was controlled using Hystar 3.2 software (Bruker, Billerica, MA, USA).

### 3.6. Linearity Range, Limit of Detection (LOD), and Limit of Quantitation (LOQ) of Standard Carnosol 

To determine the analytical range and the LOD and LOQ values, 13 solutions (1, 2, 3, 4, 5, 10, 25, 50, 100, 150, 200, 250, and 300 μg/mL) of standard carnosol (**1**) were prepared by diluting with MeOH an intermediate solution (100 μg/mL) or a stock solution (1 mg/mL) of the standard in MeOH. Each solution, after having been filtered through a syringe filter (0.22 μm pore size), was injected (0.5 μL) into the HPLC-UV-ESI-IT-MS equipment. For each dilution, three replicates were carried out, and the UV spectrum at the top of the carnosol peak in the chromatogram was recorded (Figure 3). The mean area of the absorption band with a maximum at about 285 nm was measured, and a calibration curve (y = 2.0857x − 6.7954) of concentration (μg/mL) vs. absorption area (mAU, was plotted (Figure S1 in the SI). To define the linearity range, the lowest and highest concentrations producing a %*RE* lower than 20 were selected. The limit of detection (LOD) was established as the lowest concentration that yielded a signal-to-noise ratio greater than 3. The limit of quantification (LOQ) was set as the lowest concentration within the linearity range.

### 3.7. Quantification of Carnosol in Lepechinia mutica Leaves

Three samples (250, 100, and 50 g) of dried leaves of *Lepechinia mutica* were separately extracted with EtOAc according to the procedure described in Section 3.3. After solvent evaporation, the resulting residues were pooled together to give a total of 50 g (residue A’). A sample (10 mg) of A’ was dissolved under sonication in 1.5 mL MeOH-H_2_O, 9:1 *v*/*v*, and quickly filtered through a regenerated cellulose syringe filter (0.2 μm pore size; Millipore, Bedford, MA, USA) to remove chlorophyll. A sample (5 μL) of the filtrate was injected into the HPLC-UV-ESI-IT-MS instrument. The analysis was repeated three times, and the mean concentration of carnosol = 43.1 ± 2.4 μg/mL was determined from the mean area of the UV band of (**1**), by interpolation of the calibration curve of the standard. The content C of carnosol (**1**) in the dried leaves of *L. mutica* was then calculated as 0.81 mg/g (0.081% *w*/*w*) using Equation (2):

(2) $$C\;=\frac{\mathrm{Co}\times V\times W_{A}}{W_{B}\times W}$$

where:

C: Content (mg/g) of carnosol (**1**) in the dried leaves
Co: Mean concentration of carnosol (**1**) in the dissolved sample of A’ = 43.1 · 10^−3^ mg/mL
V: Volume (1.5 mL) of MeOH-H_2_O, 9:1 *v/v*, used to dissolve the sample of A’
W_A_: Weight of total residue A’ = 50 · 10^3^ mg
W_B_: Weight of the sample of A’ from which chlorophyll was removed = 10 mg
W: Weight of dried leaves = 400 g

### 3.8. Validation of the Analytical Method

#### 3.8.1. Precision (Repeatability and Intermediate Precision)

Two analysts, 1 and 2, working independently with the same chromatographic equipment and following the procedure described in Section 3.7, determined the content of carnosol (mg/g dried leaves) in three batches (5, 10, and 15 mg) of extract A’. Each determination was carried out in triplicates in three consecutive days, and the mean, the standard deviation (SD), and the % RSD (=SD × 100/mean) were calculated for each group of experiments. The method repeatability was estimated from the data set obtained by analyst 1 (Table S1 in the SI), whereas the data sets obtained by analysts 1 and 2 (Tables S1 and S2 in the SI) were compared among themselves to estimate the intermediate precision.

#### 3.8.2. Accuracy

Accuracy was determined as the % recovery (% R) of carnosol by injecting into the HPLC-UV-ESI-IT-MS equipment samples of residue A’ (unfortified) and samples of A’ enriched (fortified) with known amounts of standard carnosol. The samples were then processed and quantified using the same analytical method mentioned above. Each sample was analyzed three times by each of the two analysts, 1 and 2. Fortified samples were prepared by adding 50 μL methanol solution of standard carnosol (700 μg/mL) to each of the three samples of A’ used in the experiments described in Section 3.8.1. The % R value was calculated for each experiment using Equation (3):

(3) $$\%\;R=\frac{\left(C_{f}-C_{u}\right)\times 100}{C_{a}}$$

where C_f_ and C_u_ are the measured concentrations (mg/g dried leaves) of carnosol (**1**) in fortified and unfortified samples, respectively, and C_a_ is the concentration of the standard carnosol (**1**) added. C_f_ and C_u_ were determined using interpolation of the calibration curve. The graphs of the % R values for the two groups of analyses are shown in Figure 5. 

#### 3.8.3. Robustness

The parameters that were changed one by one with respect to the optimized conditions were the flow rate, the column oven temperature, the UV detection wavelength, the relative proportions of acetonitrile, water, and formic acid in mobile phases A and B, and the gradient program (Table 1). In each experiment, the resolution peak between rosmarinic acid and carnosol was determined using Equation (4):

(4) $$R=\frac{\left(Tr_{2}-Tr_{1}\right)}{\frac{1}{2}(W_{1}+W_{2})}$$

where:

R: Peak resolution
*Tr*_1_: Retention time (min) of the compound that elutes first (rosmarinic acid)
*Tr*_2_: Retention time (min) of the compound that elutes second (carnosol)
W_1_: Peak width (min) of rosmarinic acid
W_2_: Peak width (min) of carnosol

Subsequently, the %RSD was calculated for each parameter changed.

### 3.9. Statistical Analysis

All parameters were evaluated in an Excel spreadsheet. For repeatability and reproducibility data, an analysis of variance (ANOVA) test was performed to determine the coefficient of variation and the *F*-value.

## 4. Conclusions

In summary, we developed a simple and accurate HPLC-UV-ESI-IT-MS method for the dosage of bioactive diterpenoid carnosol (**1**) in an EtOAc extract of *Lepechinia mutica* (Lamiaceae), a medicinal plant endemic to Ecuador. The procedure complied with all the main parameters recommended by the IUPAC and ICH guidelines [23,24] to validate an analytical method. The content of carnosol (**1**) in dried leaves was estimated to be 0.081% *w*/*w*, which was significantly higher than the yield of the same compound isolated using column chromatography (see Section 3.3). This discrepancy could depend on several factors; however, it indicated that the quantitative isolation of carnosol (**1**) from *L. mutica* would require an improved procedure. Nevertheless, the high content of carnosol (**1**) in the leaves indicated that extracts of *L. mutica* are potentially interesting sources of pure carnosol for future biological and pharmacological studies.

In comparison with *L. mutica*, *Salvia officinalis* was found to contain 4.3% carnosol [11], while the content of carnosol in dried leaves of *Rosmarinus officinalis* varied from 0.2% [31] to 4.6% [32]. The high variability of the carnosol content in the same species may depend on the genetic origin, as well as different seasonal, geographical, and agronomic parameters. On the other hand, carnosol (**1**) is considered the major oxidized product of diterpenoid carnosic acid (**2**), and the high concentrations of carnosol in the extracts are considered to indicate a plant exposed to stress conditions, as well as an aged or badly manipulated sample [31,33]. In this regard, it is interesting to note that, although not yet quantified, carnosic acid (**2**) appeared to be much more abundant than carnosol (**1**) in the extract of *L. mutica* (Figure 4). 

In the future, we shall apply our analytical procedure to quantify carnosol (**l**) in the extracts of other *Lepechinia* species as well as taxa of other genera belonging to the Lamiaceae family. 

## Figures and Tables

**Table 1:** Changes in the parameters used to evaluate the robustness of the analytical method.

Parameter		Resolution	RSD, %
Flow (mL/min)	0.2	0.77 ± 0.05	4.5
	0.4	0.74 ± 0.01	
UV detection wavelength (nm)	278	0.78 ± 0.01	2.7
	283	0.77 ± 0.00	
	290	0.78 ± 0.04	
Oven temperature (°C)	18	0.82 ± 0.01	4.6
	20	0.81 ± 0.01	
	25	0.79 ± 0.07	
Mobile phase composition ^a^	A: 27/73/0.1; B: 56/44/0.1	0.70 ± 0.04	4.9
	A: 29/71/01; B: 58/42/01	0.74 ± 0.02	
	A: 31/69/01; B: 62/38/01	0.70 ± 0.03	
Gradient program ^b^	1	0.76 ± 0.03	3.6
	2	0.79 ± 0.02	

^a^ A,B: MeCN/H_2_O/HCO_2_H; ^b^ Gradient program: (1) 100% A, from 0 to 6 min; followed by 48% A/52% B, from 6 to 17 min; followed by 100% B, from 17 to 20 min; followed by 100% A, from 20 to 25 min; (2) 100% A, from 0 to 5 min; followed by 52% A/48% B, from 5 to 20 min; followed by 100% B, from 20 to 23 min; followed by 100% A, from 23 to 28 min.

## Data Availability

All data presented in this study are available in the article and in Supplementary Materials.

## Supplementary Materials

The following supporting information can be downloaded at https://www.mdpi.com/article/10.3390/molecules28186701/s1: Figure S1. Calibration curve of standards carnosol; Table S1. Analytical data used to measure the repeatability of the analytical method; Table S2. Analytical data used to measure the intermediate precision of the analytical method.
