# Supplementary material for: A Validated HPLC-UV-ESI-IT-MS Method for the Quantification of Carnosol in Lepechinia mutica, a Medicinal Plant Endemic to Ecuador
Source: Molecules. 2023 Sep 19;28(18):6701. doi: 10.3390/molecules28186701 (PMC10536308; doi:10.3390/molecules28186701)
Supplement: Supplementary file 1 [file molecules-28-06701-s001.zip › molecules-2603516-supplementary.pdf]

SUPPLEMENTSRY INFORMATION

A validated HPLC-UV-ESI-IT-MS method for the quantification of carnosol in *Lepechinia mutica*, a medicinal plant endemic to Ecuador

Natalí Solano-Cueva [1], Jorge G. Figueroa [1], Corina Loja [1], Chabaco Armijos [1], Giovanni Vidari [2] and Jorge Ramírez [1,*]

[1] Departamento de Química, Universidad Técnica Particular de Loja, 1101608 Loja, Ecuador; nesolano@utpl.edu.ec (N.S.-C.); jgfigueroa@utpl.edu.ec (J.G.F.); cbloja@utpl.edu.ec (C.L.); cparmijos@utpl.edu.ec (C.A.)
[2] Medical Analysis Department, Faculty of Science, Tishk International University, Erbil 44001, Iraq; vidari@unipv.it
* Correspondence: jyramirez@utpl.edu.ec

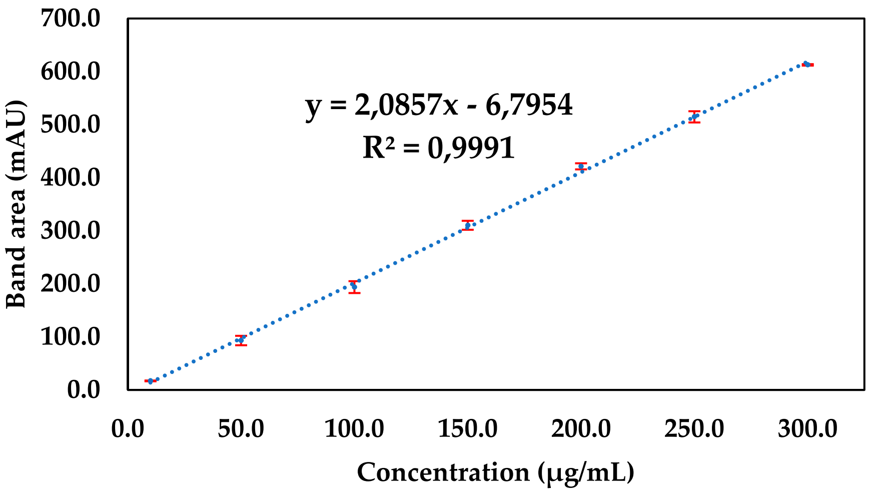

Figure S1. Calibration curve of standard carnosol (**1**). Error bars show the standard deviation of the mean.

Table S1. Analytical data, obtained by analyst 1, that were used to measure the repeatability of the analytical method.

Weight of residue A′ (mg)	Carnosol concentration (mg/g dry leaves)			RSD (%)
	Day 1	Day 2	Day 3	
5	0.84	0.79	0.78	3.6
10	0.80	0.84	0.79	3.3
15	0.83	0.80	0.79	2.8

RSD: Relative standard deviation.

Table S2. Analytical data, obtained by analyst 2, that were used to measure the intermediate precision of the analytical method.

**Weight of residue A′ (mg)**	**Carnosol concentration (mg/g dry leaves)**			**RSD (%)**
	**Day 1**	**Day 2**	**Day 3**	
5	0.83	0.78	0.83	3.6
10	0.81	0.83	0.78	3.2
15	0.79	0.81	0.82	1.9

RSD: Relative standard deviation.
